# Information for the Patient and Dentist

**Published:** 1898-12

**Authors:** 


### Bibliography.

Information for the Patient and Dentist. A Magazine for the Reception-Room Table. Published monthly by L. P. Bethel, Kent, Ohio, November, 1898.

This is the first number of a somewhat novel publication to educate "the public in regard to the better care of these important organs, the teeth.

The proper education of the public in regard to the care of the teeth has been one of the most important duties of the practising dentist. The lessons have been only partially learned, and the majority of people to-day regard the dentist as a last resort for the relief of pain. Various methods have been resorted to to bring about a better state of things, and this attempt of Dr. Bethel is warmly welcomed as a means to that end.

The present number opens with an article by Dr. Case. This is in line with his well-known and valuable work; but the question arises whether it is not too abstruse for the ordinary lay reader? The need is for short articles, free from technicalities, showing the importance of attention to the teeth from the earliest period to the final development of the permanent series. This is the object of the editor, and it is believed he will faithfully carry it out. This first number contains several articles of special value in this direction.

The publication can be warmly welcomed, and it should be upon the table of every reception-room for the education of both young and old.

